# Properties of Carotenoids in Fish Fitness: A Review

**DOI:** 10.3390/md18110568

**Published:** 2020-11-19

**Authors:** Toshiki Nakano, Geert Wiegertjes

**Affiliations:** 1 Marine Biochemistry Laboratory, International Education and Research Center for Food and Agricultural Immunology, Graduate School of Agricultural Science, Tohoku University, Sendai 980-8572, Japan; 2 Aquaculture and Fisheries Group, Wageningen University and Research, 6708 WD Wageningen, The Netherlands; geert.wiegertjes@wur.nl

**Keywords:** carotenoids, astaxanthin, canthaxanthin, reactive oxygen species, immune system, oxidative stress, antioxidant, antibiotics, thermal stress, disease

## Abstract

Carotenoids, one of the most common types of natural pigments, can influence the colors of living organisms. More than 750 kinds of carotenoids have been identified. Generally, carotenoids occur in organisms at low levels. However, the total amount of carotenoids in nature has been estimated to be more than 100 million tons. There are two major types of carotenoids: carotene (solely hydrocarbons that contain no oxygen) and xanthophyll (contains oxygen). Carotenoids are lipid-soluble pigments with conjugated double bonds that exhibit robust antioxidant activity. Many carotenoids, particularly astaxanthin (ASX), are known to improve the antioxidative state and immune system, resulting in providing disease resistance, growth performance, survival, and improved egg quality in farmed fish without exhibiting any cytotoxicity or side effects. ASX cooperatively and synergistically interacts with other antioxidants such as α-tocopherol, ascorbic acid, and glutathione located in the lipophilic hydrophobic compartments of fish tissue. Moreover, ASX can modulate gene expression accompanying alterations in signal transduction by regulating reactive oxygen species (ROS) production. Hence, carotenoids could be used as chemotherapeutic supplements for farmed fish. Carotenoids are regarded as ecologically friendly functional feed additives in the aquaculture industry.

## 1. Introduction

It is well known that fish have a high risk of exposure to various environmental stressors and infectious diseases. Accordingly, it is thought that effective supplements, such as probiotics, vaccines, vitamins, peptides, lipids, nucleotides, and disease treatments, such as antibiotics, are needed for successful aquaculture [1,2,3,4,5,6,7,8,9,10,11,12,13,14,15]. Infectious diseases are one of the primary contributors to economic loss in the aquaculture industry [10,16]. Antibiotics have been used to prevent disease and improve feed efficiency in fish [17,18,19]. However, the use of antibiotics in aquaculture entails numerous problems, such as the generation of antibiotic-resistant bacteria, accumulation of residual antibiotics in fish tissues, and side effects on fish health [20]. The prevention of disease is always a priority over medicinal treatment. Therefore, functional feed ingredients from natural sources might be considered as safe agents for diseases prevention in farmed animals and humans.

Carotenoids are lipid-soluble pigments that have conjugated double bonds and are the most common class of pigments that occur in nature. More than 750 kinds of carotenoids have been identified. Carotenoids show robust antioxidative activity and have been widely used in the pharmaceutical, cosmetic, food, and feed industries owing to their biological characteristics without exhibiting any cytotoxicity or side effects [21,22,23,24,25,26,27,28,29,30,31]. Through epidemiological studies and clinical trials, dietary carotenoids in mammals have been found to have a correlation with several positive biological effects. Furthermore, carotenoids have several physiological effects, such as those on the immune system, reproduction, lipid metabolism, photoprotection in skin, diseases such as adiposity, obesity, diabetes, cardiovascular disease, hypertension, atherosclerosis, and cancer, and inflammation [7,11,23,26,28,29,30,31,32,33,34,35,36,37,38,39,40,41,42,43,44,45,46,47,48,49,50,51,52,53,54,55,56,57,58,59,60,61,62]. Recently, the global market for carotenoids has been estimated to exceed $1.5 billion USD [63,64].

This brief review summarizes information concerning the biological properties of carotenoids, particularly astaxanthin (ASX), as a functional feed ingredient and supplement in fish. Carotenoids have attracted considerable interest and are expected to be economically valuable in aquaculture. This is because the demand for and production of farmed fish is increasing due to the worldwide decline in ocean fisheries stocks [65]. Improvements in global fish farming could relieve pressure on ocean fisheries and support the global food supply.

## 2. Sources of Carotenoids

The most common carotenoids are 40-carbon isoprenoids, which contain 11 conjugated double bonds. Carotenoids impart color to living organisms such as plants, birds, fish, crustaceans, and bacteria. Generally, carotenoids occur at low levels in organisms; however, the total amount of carotenoids in nature has been estimated to be more than 100 million tons. [7,21,23,39].

**Figure 1:**
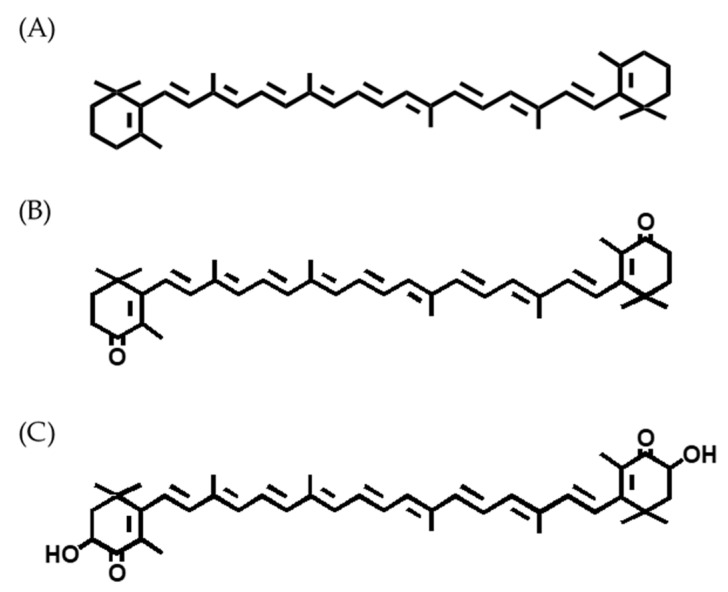
Structure of specific carotenoids in fish. (**A**) β-Carotene, (**B**) canthaxanthin, (**C**) astaxanthin. Carotenoids are classified into carotenes (e.g., β-carotene), which are composed of carbon and hydrogen, and xanthophylls (e.g., canthaxanthin and astaxanthin), which also contain oxygen.

Carotenoids are classified into carotenes (e.g., α-carotene, β-carotene, and lycopene), which are composed of carbon and hydrogen, and xanthophylls (e.g., zeaxanthin, lutein, canthaxanthin “CAX”, and ASX), which also contain oxygen [21,22,25,66,67,68]. Figure 1 shows the structure of specific carotenoids found in fish and shellfish. Most animals cannot produce carotenoids de novo. On the contrary, photosynthetic organisms, such as plants, can produce carotenoids de novo. Salmonids cannot synthesize ketocarotenoids, such as ASX (3,3′-dihydroxy-β, β-carotene-4,4′-dione). The amount of carotenoids deposited in fish muscle depends on the level of carotenoids in the feed [7,21,23]. The ASX level in wild salmonid’s muscle reportedly ranges from 3 to 38 mg/kg [27]. Accordingly, salmonid’s flesh seems to be a good dietary source of ASX.

Many carotenoids, in particular ASX, have been applied to coloration of farmed fish [7,11,27,44,69,70,71,72,73,74]. ASX is a common carotenoid used to enhance the pigmentation of farmed salmon. Enhanced salmon filet pigmentation is a vital quality parameter, in that it can influence the consumer’s decision for purchasing. The sources of carotenoids used in aquaculture are synthetic ASX, such as CAROPHYLL Pink (DSM Nutritional Products Ltd., Kaiseraugst, Switzerland), green microalgae *Haematococcus pluvialis*, red yeast *Xanthophyllomyces dendrorhous* (formerly designated *Phaffia rhodozyma*), bacteria *Paracoccus carotinifaciens*, krill oil and meal, crayfish oil, etc. The sources of carotenoids mentioned above are summarized in Table 1 [7,11,26,27,58,62,70,73,75,76,77,78,79,80]. The level of ASX in red yeast, krill, and crayfish is low. In contrast, *H. pluvialis* accumulates high levels of ASX, which can accumulate up to 7% of the dry weight of the alga during its growth in response to stressful cultural conditions under intense sunlight [62,64,78,80,81].

**Figure 2:**
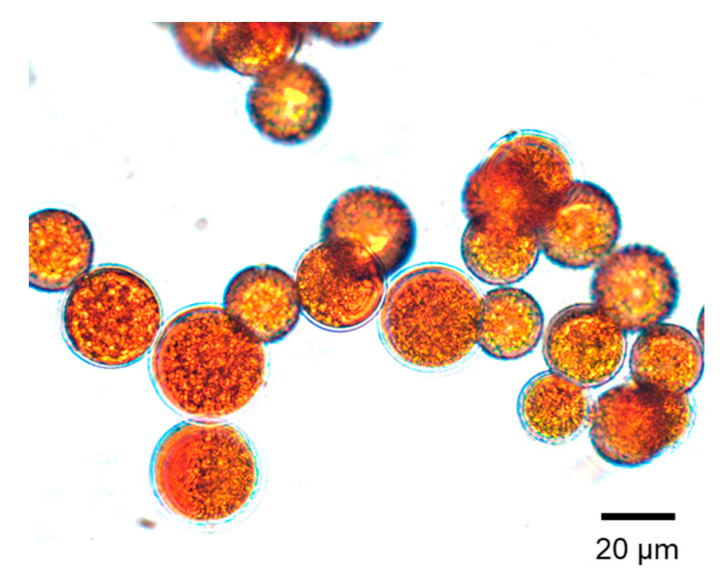
Picture of *Haematococcus* sp. *Haematococcus* produces and accumulates high levels of astaxanthin under conditions of intense sunlight. Credit: Dr. G. Nishitani.

The microscopic picture of *Haematococcus* sp. that accumulated plenty of ASX in their cells is shown in Figure 2. ASX is known to be usually esterified with fatty acid in microalgae [58]. ASX in *Haematococcus* contains about 70% of monoesters type, 25% of diesters type, and 5% of non-ester-free type [75]. The major manufacturers which produce ASX from *Haematococcus* are in the United States (Hawaii), Japan, Israel, and Canada [81].

## 3. Activities of Carotenoids in Farmed Fish

Besides having clear effects on coloration, carotenoids, such as ASX, are considered semi-essential nutrients that promote optimal survival and growth at relatively low dietary-inclusion levels. Several studies have shown that growth or survival can be significantly improved when fish are fed a diet with carotenoids compared to when they are fed a diet without carotenoids [7,11,27,30,44,46,70]. Generally, ASX has been recommended to use in the level of 50–100 mg/kg diet to enhance muscle pigmentation in fish [82]. Furthermore, greater attention needs to be given to carotenoid biotransformation and absorption, and the detection of carotenoid metabolites in the body [26,58,83]. Several analytical methods have been utilized to investigate the role of carotenoids in the body. For example, high-performance liquid chromatography (HPLC), mass spectrometry (MS), and nuclear magnetic resonance (NMR) have been applied for the detection of carotenoid metabolites [24,25,31,68,84]. In particular, LC time-of-flight MS/MS (LC-TOFMS/MS) is believed to be a useful analytical method for natural chemicals and biological compounds, such as carotenoids [85,86].

As shown in Table 2, carotenoids are reported to have many excellent functions in fish [1,7,8,11,27,44,45,46,70,74,87,88,89,90,91]. In the following sections, the biological effects of carotenoids, such as ASX, on fish fitness, particularly on the immune system, cellular damage, and oxidative stress, are described.

### 3.1. Effects of Important Carotenoids Such as ASX on the Fish Immune System

Carotenoids may help prevent lipid peroxidation, reduce cellular oxidative stress, and reduce inflammatory response in tissues [11,26,28,30,44,45,46,92,93,94]. In short, carotenoids can help prevent chronic stress otherwise caused by a too-strong inflammatory response as part of an overreactive immune system. How? The localized physiological response known as inflammation, in which an affected area may become reddened, swollen, hot, or even painful, is typically part of the body’s normal reaction to injury or infection. When controlled from overreactions, inflammation is and remains an essential part of the innate immune response and helps recruit histamines, white blood cells, prostaglandins, phagocytes, and other cellular mediators to an injured site. Thus, particularly at the early phase of immune responses, inflammation could be considered advantageous, whereas chronic inflammation at a later phase of immune responses could be considered stressful and in need of being prevented.

Carotenoids can convert endogenously produced reactive oxygen species (ROS), such as hydrogen peroxide (H_2_O_2_), hydroxyl radicals (OH), and superoxide anions (O_2_^-^), into more stable products that cause less damage to cell membranes. The exact dose of carotenoids is relevant and could be important, because the free radicals listed above form an essential part of the innate immune defense system and preferably their effect should only be moderated in situations of overreactions, preventing chronic inflammation. Higher-than-optimal doses of carotenoids could counteract the potentially beneficial effects of these same free radicals released during early inflammatory responses to attack bacterial membranes. After all, professional phagocytes such as neutrophilic granulocytes and macrophages are part of the innate immune system and function primarily with the help of the above-mentioned free radicals to target and destroy pathogens. Indeed, together with nitric oxide (NO), which is induced by cytokine-inducible NO-synthase (iNOS or NOS2), both ROS and reactive nitrogen species (RNS) are essential mediators of the microbicidal activity of these professional phagocytes [94,95]. Overall, the release of RNS and ROS by professional phagocytes, generally referred to as the oxidative burst, is considered an important defense mechanism designed to destroy pathogens and should, therefore, be considered a beneficial reaction that should not be annulled or interfered with by high levels of antioxidants (i.e., carotenoids). Overshoot of this reaction, in contrast, could lead to situations of chronic inflammation that could be counteracted by carotenoids: nitrogen and oxygen radicals should only be considered beneficial up to a point.

Upon overreactions of the immune system, professional phagocytes may end up over-producing nitrogen and oxygen radicals by continuous and chronic activation, contributing to cell dysfunction and cell death beyond that of bacteria. Indeed, in the presence of Fe^2+^ ions and through the Fenton reaction, H_2_O_2_ can be broken down into ·OH, which are the most reactive ROS and can also damage host cell structures (proteins, lipids, and DNA). Further, mitochondrial NO can react with O_2_- to yield peroxynitrite (ONOO−), a highly potent oxidant that is even more reactive than its precursors. This oxidant can cause nitration (mainly in tyrosine residues) of many proteins, but can also oxidize amino acid residues essential for the functioning of several proteins, including transcription factors and enzymes [94,96,97,98]. Not only in mammals but also in fish, among the professional phagocytes, macrophages appear better equipped to fight potentially damaging and, therefore, negative aspects of oxidation than neutrophils [99]. No matter what, even when produced and (mostly) contained by professional phagocytes, very high concentrations of RNS and/or ROS can be harmful and need to be countered.

As introduced above, carotenoids are classified into carotenes and xanthophylls, the latter including ASX, a major colorant of farmed fish and common carotenoid used to enhance the pigmentation of farmed salmon. Most studies on the effects of carotenoids on the immune system of fish, therefore, have been performed with ASX. In studies targeting farmed fish species, clues on innate immune regulation by ASX frequently come from studying messengers important for the immune system, such as cytokines, and mostly by measuring gene expression levels by real-time quantitative PCR, simply because practical alternatives to measure protein rather than gene expression, such as enzyme-linked immunosorbent assay (ELISA), regretfully have remained scarce [100]. Pro-inflammatory cytokines are the most studied, and most frequently, those are interleukin-1 beta (IL-1β), interleukin-6 (IL-6), and tumor necrosis factor-alpha (TNFα), also in fish [101], but the outcomes are difficult to interpret. For example, in a recent study examining effects of dietary CAX (ASX is a metabolite of CAX) on the growth performance and antioxidant status of yellow perch (*Perca flavescens*), the authors reported an upregulation of IL-1β gene expression in groups fed CAX, compared to that in control fish not receiving CAX [102]. In apparent contrast, exposure of snakehead (*Channa argus*) hepatocytes to bacterial lipopolysaccharides showed that these cells could be protected from a subsequent inflammatory response by pretreatment with ASX, preventing the upregulation of IL-1β, IL-6, and TNFα [103]. Indeed, a complicating factor in studies on the effects of external factors such as carotenoids on immune responses, is that pro-inflammatory cytokines typically can be produced by a broad range of cells that are not always exclusively immune cells. Maybe, rather than studying up- or down-regulation of a single cytokine gene, complex studies into immune-modulating effects of factors such as carotenoids are better off taking whole-transcriptome approaches [104], more broadly addressing the research question. After all, a reliable analysis of complex physiological changes requires the use of equally complex arrays of gene expression or other bio-assay results before concluding on fish health or disease resistance. Of course, this does not rule out IL-1β as an informative marker, but it does point at the complexity of studies addressing the physiological effects of carotenoids, such as ASX, on the immune system.

Although maybe difficult to explain in immunological detail without further studies on the fish immune system, it seems likely that professional phagocytes such as macrophages, along with their own cell-related antioxidant and cytoprotective pathways that help reduce the oxidative burden, can make use of diet-derived carotenoid antioxidants such as ASX, as they do for ascorbic acid and α-tocopherol [96]. Certainly, since the body concentrations of ROS scavenger molecules, such as ASX and β-carotene, are influenced by dietary intake, it is conceivable that higher concentrations of carotenoids in the diet could help professional phagocytes overcome the side effects caused by excessive oxidation, restrict chronic inflammation and therefore, be beneficial. These conclusions appear supported by studies in several fish species that report on a variety of enhanced immune reactivities after dietary supplementation of ASX and β-carotene [105,106,107,108,109,110]. Thus, like mammals [11,111], carotenoids may be regarded as biogenics with enough potential to modulate ROS/RNS production and immune function in fish.

### 3.2. Effects of Carotenoids, Particularly ASX, on Cellular Damage and Oxidative Stress in Fish

Several kinds of carotenoids have provitamin A activity, stimulate growth, and improve fish reproduction [13,27,70,112]. Salmonid eggs usually contain high ASX levels, which can enhance the quality of eggs during the initial feeding period of growth. Additionally, ASX can protect salmonid egg membranes from oxidative injury caused by UV radiation [27,54,70]. This photoprotection activity of ASX might be one of the most specific functions of carotenoids in organisms, including fish.

Recently, attention has been given to the anti-stress activity of carotenoids, besides their provitamin A activity. This is because farmed fish are often exposed to environmental stressors in ordinary cultural conditions [20,113,114,115,116,117,118,119,120,121]. Environmental stress in fish is thought to be derived from several factors (stressors), such as diseases, chemicals, acute changes in temperature, heavy metals, and aquacultural conditions.

Several cases regarding environmental stress in fish have been reported. The bacterium *Edwardsiella tarda* is a virulent intracellular pathogen found in commercial fish species. Edwardsiellosis, caused by *E. tarda*, is one of the most severe diseases in the Japanese flounder *Paralichthys olivaceus*. Heat shock protein 70 (HSP70) expression was increased in fish infected with *E. tarda*, compared to that in control fish [16]. The expression levels of Cu, Zn–superoxide dismutase (Cu, Zn–SOD) in fish increased after infection. In contrast, the expression level of Mn–SOD in fish gradually increased after infection and remained high between 24 and 48 h post-infection [16].

In aquaculture, chemicals such as antiseptics, antibiotics, and parasiticides are often used to prevent or treat diseases [19]. Oxytetracycline (OTC) is an antibiotic that belongs to the tetracycline family and has been used against several bacterial infections in farmed fish. However, high doses of OTC have been known to cause side effects in fish [17,18,19]. The glutathione (GSH) levels in the plasma, liver, muscle, and stomach of OTC-fed fish were higher than those in control fish [20]. Studies have demonstrated that organisms have both enzymatic and non-enzymatic antioxidant defense systems against ROS [98,121,122,123,124,125,126,127,128]. GSH is the major non-protein cellular thiol with reducing and nucleophilic properties [94,98,121,125,129,130]. GSH is also known to be the substrate for glutathione peroxidase (GPX), an antioxidative enzyme that can eliminate the lipid peroxide (LPO) generated within cells [94,121,127,128,130]. Accordingly, OTC-induced stress might increase the metabolic turnover of GSH due to its consumption by scavenging oxidants generated by chemical stress.

The physiological states of fish depend on their environmental temperature. Accordingly, the environmental temperature can induce numerous changes in the fish body. Increased temperature heightens oxygen consumption, resulting in increased ROS production [118,124]. The effect of thermal stressors on redox-related biomarkers in fish has been reported [118]. For example, the plasma LPO levels in fish exposed to heat shock were shown to increase. LPO in stressed fish plasma might be derived from various damaged tissues. The plasma GSH levels in fish initially decreased but returned to basal levels at 17.5 h post-heat shock [118]. Considered one of the primary antioxidative enzymes, the plasma activity of SOD in stressed fish was significantly increased compared with that in the control group after heat shock. Moreover, the expression levels of hepatic GSH and HSP70 gradually increased after heat shock treatment. HSP70 assists in the folding of nascent polypeptide chains, acts as a molecular chaperon or helper molecule, mediates the repair and degradation of denatured proteins, and is involved in the breakdown and replacement of irreparable proteins. HSPs have various species-specific functions and are usually induced during stress. In response to stressors, such as chemicals, heat shock, bacterial pathogens, pollutants, and physiological states, the induction of various HSP families has already been reported in cell lines and various fish tissues [113,115,118,120,131,132,133,134,135,136].

The fish oil used in aquafeed contains plenty of highly unsaturated fatty acids (HUFA), such as eicosapentaenoic acid (EPA) and docosahexaenoic acid (DHA), which are readily oxidized. The levels of serum LPO, transaminase activity, and lipids (triglycerides, total cholesterol, and phospholipids) in fish fed oxidized oil were observed to be higher than those in fish provided non-oxidized oil [46]. Oxidized oil usually contains LPO and secondary products. LPO is further decomposed into a variety of ROS that attack cell components and induce stress [94,96,98,122,125,137].

Hence, the above-mentioned results concerning the changing patterns of stress-related biomarkers, such as GSH, SOD, LPO, and HSP70, suggest that the stressors should affect the redox state and induce oxidative stress in fish. These results also suggest that GSH, SOD, and HSP70 might play essential roles in mediating the response and the defense upon oxidative stress in fish. The production of ROS, which are strong oxidants and induce oxidative stress in cells, is already known to increase under certain stressful conditions. The oxidative stress often leads to an increased risk of diseases [35,38,42,46,94,98,118,121,122,123,125,137,138,139,140,141,142]. Accordingly, insufficient ingestion of antioxidants, such as carotenoids and α-tocopherol, might increase an organism’s susceptibility to oxidative stress-related diseases.

The biological effects of carotenoids, such as ASX and lycopene, on oxidative stress in fish have been reported [7,11,27,30,44,45,46,74,107,142,143]. The liver and plasma from fish fed ASX were observed to have a significantly higher level of α-tocopherol and carotenoids than those from control fish fed a non-ASX diet. On the contrary, ASX significantly decreased LPO levels in several tissues. Plasma LPO is a useful indicator of tissue damage due to oxidative stress in the body [144]. ASX, including red yeast, was also found to decrease the level of LPO in fish serum [30,44,46]. Most LPO is found in lipoproteins that contain highly susceptible lipids [54,144]. ASX and α-tocopherol exist in the lipoproteins of fish serum along with several kinds of circulating antioxidants [30,145]. Carotenoids and α-tocopherol are thought to be in biological membranes, which contain a large amount of HUFA. Hence, ASX, α-tocopherol, and other antioxidants might synergistically protect lipoproteins from oxidation, thereby reducing oxidative stress in the body. Additionally, the administration of carotenoids, such as ASX and lycopene, have been observed to enhance the production of antioxidative enzymes, such as SOD and GPX, and the cellular endogenous antioxidants, such as GSH, in fish, mammals, and invertebrate [27,37,43,74,107,142,143,146,147]. The supplementation of carotenoids has also been found to upregulate the expression of HSP in the tissue of several animal species and cultured cells [27,107,146,148,149]. Taken together, these results suggest that dietary carotenoids can improve the antioxidative defense including antioxidative enzymes, cellular endogenous antioxidants, and HSP, and strengthen the ability of resistance against oxidative stress in the body.

ASX is known to be one of the most effective antioxidative compounds owing to its specific structure. Indeed, the antioxidative activity of ASX is 100-fold and 10-fold higher than that of α-tocopherol and β-carotene, respectively [62,150]. ASX has conjugated carbon-carbon double bonds and hydroxyl (–OH) and keto (=O) groups in its structure and shows both lipophilic (hydrophobic, non-polar) and hydrophilic (polar) properties. The hydroxyl groups of ASX are anchored across membranes, with polar functional groups oriented outside the membrane. The backbones of ASX might serve as a molecular wire increasing the mechanical strength of the membrane [28,38,49,151]. Accordingly, membrane-bound antioxidants, such as ASX and α-tocopherol, protect membrane lipids and proteins from oxidation. Carotenoids can improve the cellular antioxidant capacity by regenerating α-tocopherol and ascorbic acid from their corresponding radical forms [94,152]. Carotenoids show cooperative or synergistic interactions with other antioxidants such as α-tocopherol, ascorbic acid, and GSH, located in lipophilic and hydrophobic compartments of tissue [37]. Essentially, among trout fibroblastic cells cultured under ROS-induced oxidative stress, membrane damage was observed in cells without ASX, whereas cells containing ASX had suppressed cellular membrane damage and increased viability [30]. These results suggest that ASX can effectively protect cells against oxidative damage induced by ROS and maintain both membrane dynamics and cellular function.

### 3.3. Molecular Mechanisms of Carotenoid’s Actions in the Body

The antioxidative activity shown by carotenoids might not only act by directly scavenging ROS, but also by modulating expression of stress and antioxidative-related proteins. The molecular mechanisms of action shown by carotenoids in the body have been classified into the following four categories: (1) antioxidative and pro-oxidative activities, (2) suppression of the nuclear factor kappa-light-chain-enhancer of activated B cells (NF-κB) signaling translation, (3) activation of the nuclear factor erythroid 2-related factor 2 (Nrf2), and (4) interaction with other transcription factors [26,142,153,154]. Transcription factors such as NF-κB and Nrf2 are known to associate with immune reaction, inflammation, and oxidative stress responses. The NF-κB pathway is activated by inflammatory compounds, such as tumor necrosis factor-α (TNF-α) and cytokines, and oxidative stress. On the contrary, the Nrf2 pathway is known to be an important pathway in the defense against ROS-induced oxidative stress in cells [153]. The conjugated double bond in carotenoids can act as a strong antioxidant by donating electrons and reacting with free radicals to convert them to more stable forms. Carotenoids are thought to react with free radicals through the following three reaction mechanisms: (1) electron transfer (oxidation and reduction), (2) hydrogen abstraction (allylic hydrogen atom abstraction), and (3) radical addition (adduct formation) [37,43,49,54,80,94,155,156,157]. In addition, intercellular signaling is often affected by ROS and oxidative stress, resulting in changes in the expression of several genes [122,124,125,129,139,158,159,160].

Accumulated ROS potentially activate signaling cascades involving transcription factors such as NF-κB and activator protein-1 (AP-1), and induce the expression of SODs to neutralize the harmful effects of ROS [74,118,124,158,161,162,163,164,165]. ASX can inhibit ROS-induced expressions of NF-κB, which is involved in the transcription of inducible genes, such as heme oxygenase 1 (HO-1) and iNOS, that regulate inflammatory responses and oxidative stress [58,153]. ROS-induced oxidative stress has been reported to activate several kinases, such as protein kinase A (PKA), mitogen-activated protein kinase (MAPK), c-Jun N-terminal kinase, extracellular signal-regulated kinase, and transcription factor AP-1 [166]. The accumulation of Nrf2 protein can induce the expression of HO-1, and the phosphorylation of EPK and p38 kinase [58]. Oxidative stress is also known to attenuate post-PKA pathways, resulting in inhibited expression of the mature form of steroidogenic acute regulatory protein (STAR). ASX can prevent the downregulation of the expression of STAR caused by ROS [43,58,167]. Thus, under oxidative stress, carotenoids might modulate gene expression accompanying alterations in signal transduction through the regulating ROS formation in the tissue.

## 4. Conclusions

The demand for and production of farmed fish has increased due to the worldwide decline of ocean fisheries stocks. It is known that almost half of the world’s fishery production is currently based on aquaculture [65]. The total global aquaculture production is estimated to have reached about 82 million tons of aquatic animals ($250 billion USD) in 2018. The aquatic animals farming was dominated by finfish (54 million tons) in 2018 [65]. Aquaculture could be a major source of aquatic dietary proteins by 2050 as it can sustainably enhance food supply. Thus, appropriate monitoring and evaluation methods for impact and risk of aquaculture on the environment need to be considered [65,168,169,170,171]. Additionally, Atlantic salmon (*Salmo salar*), rainbow trout (*Oncorhynchus mykiss*), and coho salmon (*Oncorhynchus kisutch*) are known to be valued salmonid species used in aquaculture worldwide [65,85,114,172,173,174]. Particularly in Japan, coho salmon farming is one of the basic industries in northeastern Pacific coastal areas where the great earthquake and tsunami occurred in 2011 [173,175,176]. Accordingly, there is a desire to improve productivity of coho salmon farming and quality of coho salmon for reconstruction of coastal fisheries in disaster-stricken areas.

Many substances have been used for farmed fish as feed supplements. Carotenoids, being one of them, should be safe for fish and fish consumers. The use of carotenoids in aquaculture should employ an ecologically friendly method. Thus, carotenoids seem to be one of the ideal functional ingredient types in aquaculture. However, some carotenoids have been suggested to act as pro-oxidants under certain conditions [26,28,43,155,156,157,177,178]. It is important to find and maintain an appropriate balance between antioxidants and oxidants in the body [26,34,156,179,180,181,182,183]. In addition, carotenoids have been known to be susceptible to oxidation, so that peroxides perhaps could occur in carotenoid samples during storage. These peroxides might influence the actions of carotenoids as a pro-oxidant [28,156]. Further studies are required to reveal the relationships between antioxidation, oxidative stress, immune stimulation, and gene expression modulated by carotenoids, and their contribution to fish fitness. These studies would provide useful information to establish sustainability in aquaculture.

## Figures and Tables

**Table 1:** Sources of carotenoids in farmed fish.

Source
Synthetic astaxanthin (CAROPHYLL Pink, DSM Nutritional Products Ltd.)
Synthetic canthaxanthin (CAROPHYLL Red, DSM Nutritional Products Ltd.)
Red yeast *Xanthophyllomyces dendrnrhous* (formerly designated *Phaffia rhodozyma*)
Green microalgae *Haematococcus pluvialis*
Bacteria *Paracoccus carotinifaciens*
Krill *Euphausia superba*
Alga *Spirulina* spp.
Crustacean exoskeleton and meal
Red pepper paprika *Capsicum annuum* L
Marigold

**Table 2:** General biological functions of carotenoids in fish.

Biological Function
Antioxidant activity
Anti-stress
Anti-inflammatory
Egg quality
Growth performance
Immune system
Lipid metabolism
Liver function
Muscle pigmentation
Photoprotection
Provitamin A activity
Reproduction
